# Effect of liposomal bupivacaine for preoperative erector spinae plane block on postoperative pain following video-assisted thoracoscopic lung surgery: a protocol for a multicenter, randomized, double-blind, clinical trial

**DOI:** 10.3389/fmed.2024.1359878

**Published:** 2024-04-12

**Authors:** Dawei Liao, Ke Peng, Yang Zhang, Huayue Liu, Zhongyuan Xia, Jian Guo, Fujiang Wei, Chen Chen, Xin Lv, Jianhua Tong, Xiaoshuang Li, Xianfeng Qu, Xiaobin Wang, Yingbin Wang, Shanshan Ou, Hong Liu, Xisheng Shan, Fuhai Ji

**Affiliations:** ^1^Department of Anesthesiology, The First Affiliated Hospital of Soochow University, Suzhou, China; ^2^Institute of Anesthesiology, Soochow University, Suzhou, China; ^3^Department of Anesthesiology, Tongren People's Hospital, Tongren, China; ^4^Department of Anesthesiology, Renmin Hospital of Wuhan University, Wuhan, China; ^5^Department of Anesthesiology, The Fourth Affiliated Hospital Zhejiang University School of Medicine, Yiwu, China; ^6^Department of Anesthesiology, Yantaishan Hospital, Yantai, China; ^7^Department of Anesthesiology, The First People’s Hospital of Changzhou, Changzhou, China; ^8^Department of Anesthesiology, Shanghai Pulmonary Hospital, Shanghai, China; ^9^Department of Anesthesiology, The Second Affiliated Hospital of Nanjing Medical University, Nanjing, China; ^10^Department of Anesthesiology, Lianshui County People's Hospital, Huaian, China; ^11^Department of Anesthesiology, Taizhou Municipal Hospital, Taizhou, China; ^12^Department of Anesthesiology, The Affiliated Hospital of Southwest Medical University, Luzhou, China; ^13^Department of Anesthesiology, Lanzhou University Second Hospital, Lanzhou, China; ^14^Department of Anesthesiology, The Fifth Affiliated Hospital of Sun Yat-sen University, Zhuhai, China; ^15^Department of Anesthesiology and Pain Medicine, University of California Davis Health, Sacramento, CA, United States

**Keywords:** liposomal bupivacaine, erector spinae plane block, thoracoscopic, postoperative pain, area under the curve

## Abstract

**Background:**

There is still a controversy about the superiority of liposomal bupivacaine (LB) over traditional local anesthetics in postoperative analgesia after thoracic surgery. This study aims to determine the effect of LB versus bupivacaine hydrochloride (HCl) for preoperative ultrasound-guided erector spinae plane block (ESPB) on postoperative acute and chronic pain in patients undergoing video-assisted thoracoscopic lung surgery.

**Methods:**

This multicenter, randomized, double-blind, controlled trial will include 272 adult patients scheduled for elective video-assisted thoracoscopic lung surgery. Patients will be randomly assigned, 1:1 and stratified by site, to the liposomal bupivacaine (LB) group or the bupivacaine (BUPI) HCl group. All patients will receive ultrasound-guided ESPB with either LB or bupivacaine HCl before surgery and patient-controlled intravenous analgesia (PCIA) as rescue analgesia after surgery. The numeric rating scale (NRS) score will be assessed after surgery. The primary outcome is the area under the curve of pain scores at rest for 0–72 h postoperatively. The secondary outcomes include the total amount of opioid rescue analgesics through 0–72 h postoperatively, time to the first press on the PCIA device as rescue analgesia, the area under the curve of pain scores on activity for 0–72 h postoperatively, NRS scores at rest and on activity at different time points during the 0–72 h postoperative period, Quality of Recovery 15 scores at 72 h after surgery, and NRS scores on activity on postsurgical day 14 and postsurgical 3 months. Adverse events after the surgery are followed up to the postsurgical day 7, including postoperative nausea and vomiting, fever, constipation, dizziness, headache, insomnia, itching, prolonged chest tube leakage, new-onset atrial fibrillation, severe ventricular arrhythmia, deep venous thrombosis, pulmonary embolism, pulmonary atelectasis, cardiac arrest, ileus, urinary retention, chylothorax, pneumothorax, and organ failure. Analyzes will be performed first according to the intention to treat principle and second with the per-protocol analysis.

**Discussion:**

We hypothesize that LB for preoperative ultrasound-guided ESPB would be more effective than bupivacaine HCl in reducing postoperative pain in video-assisted thoracoscopic lung surgery. Our results will contribute to the optimization of postoperative analgesia regimens for patients undergoing video-assisted thoracoscopic lung surgery.

**Clinical trial registration:**http://www.chictr.org.cn, identifier ChiCTR2300074852.

## Introduction

Thoracoscopic surgery has emerged as the backbone of surgical procedures for lung resection in recent years, but these procedures still cause moderate-to-severe postoperative acute and chronic pain (1–4). Preemptive analgesia is regarded as a proven approach to minimize postsurgical pain (5). Several preemptive analgesia approaches have been employed in thoracoscopic surgery, including paravertebral block, intercostal nerve block, and thoracic epidural block (6). The erector spinae plane block (ESPB), a new inter-fascial plane block technique first described in 2016 for thoracic pain treatment (7), has the advantages of easy handling, high safety, and a good analgesic effect (8, 9). However, ESPB with a single dosage of currently available local anesthetics is limited by the short duration of analgesia (typically 24 h or less). The duration of analgesia can be prolonged by continuous peripheral nerve blocks using a perineural catheter; however, this may result in a variety of inconveniences and side effects, such as management complexity, catheter-related infections, leakage, and accidental dislocation (10, 11).

Liposome bupivacaine (LB) is a novel local anesthetic with water-soluble bupivacaine wrapped in a liposome, allowing for a steady, continuous release of the drug for up to 72 to 96 h (12, 13). In 2011, liposomal bupivacaine was approved by the U.S. Food and Drug Administration to be used in single-dose wound infiltration for postsurgical analgesia in adults, and then the indication was expanded to transverse abdominis plane blocks and interscalene brachial plexus blocks (14, 15). Liposome bupivacaine appears safe when used in fascial plane blocks and peripheral nerve blocks (16–20). Whether LB is superior to traditional local anesthetics in postoperative analgesia after thoracic surgery remains controversial. Several retrospective studies have suggested that LB applied in minimally thoracic surgery improved postoperative pain, decreased opioid use, and shortened hospital stay in comparison with bupivacaine hydrochloride (HCl) (21–23). A recent randomized controlled trial including 50 patients undergoing minimally invasive lung surgery indicated that LB for intercostal nerve block provided no benefit in mitigating postoperative pain compared with bupivacaine plus epinephrine (18). However, that study had a smaller sample size and did not exclude the patients with more complex pain problems. To date, the analgesic efficacy of LB used in ESPB procedures on acute or chronic postsurgical pain remains unknown.

In this context, we designed this multicenter randomized controlled trial to determine the effect of LB versus bupivacaine HCl for preoperative ultrasound-guided ESPB on postoperative acute and chronic pain in patients undergoing video-assisted thoracoscopic lung surgery. In addition, we will also evaluate its safety profile when used in the ESPB procedures.

## Methods

### Ethics and registration

The study protocol was approved by the ethics committees of the leading center (the First Affiliated Hospital of Soochow University, Suzhou; Approval No. 2023–207) and each participating center. This trial was registered at the Chinese Clinical Trial Registry (http://www.chictr.org.cn, identifier: ChiCTR2300074852) before the enrollment of the first subject. This protocol adheres to the guidelines of Standard Protocol Items: Recommendations for Interventional Trials (SPIRIT) statement (24).

### Study design and status

This investigator-initiated, multicenter, randomized, double-blind, parallel-controlled trial will be conducted at 12 tertiary hospitals in China. Subjects will be randomized to receive either liposomal bupivacaine or bupivacaine HCl for ultrasound-guided ESPB after induction of anesthesia. Group allocation is performed by the online central randomization system.

**Figure 1:**
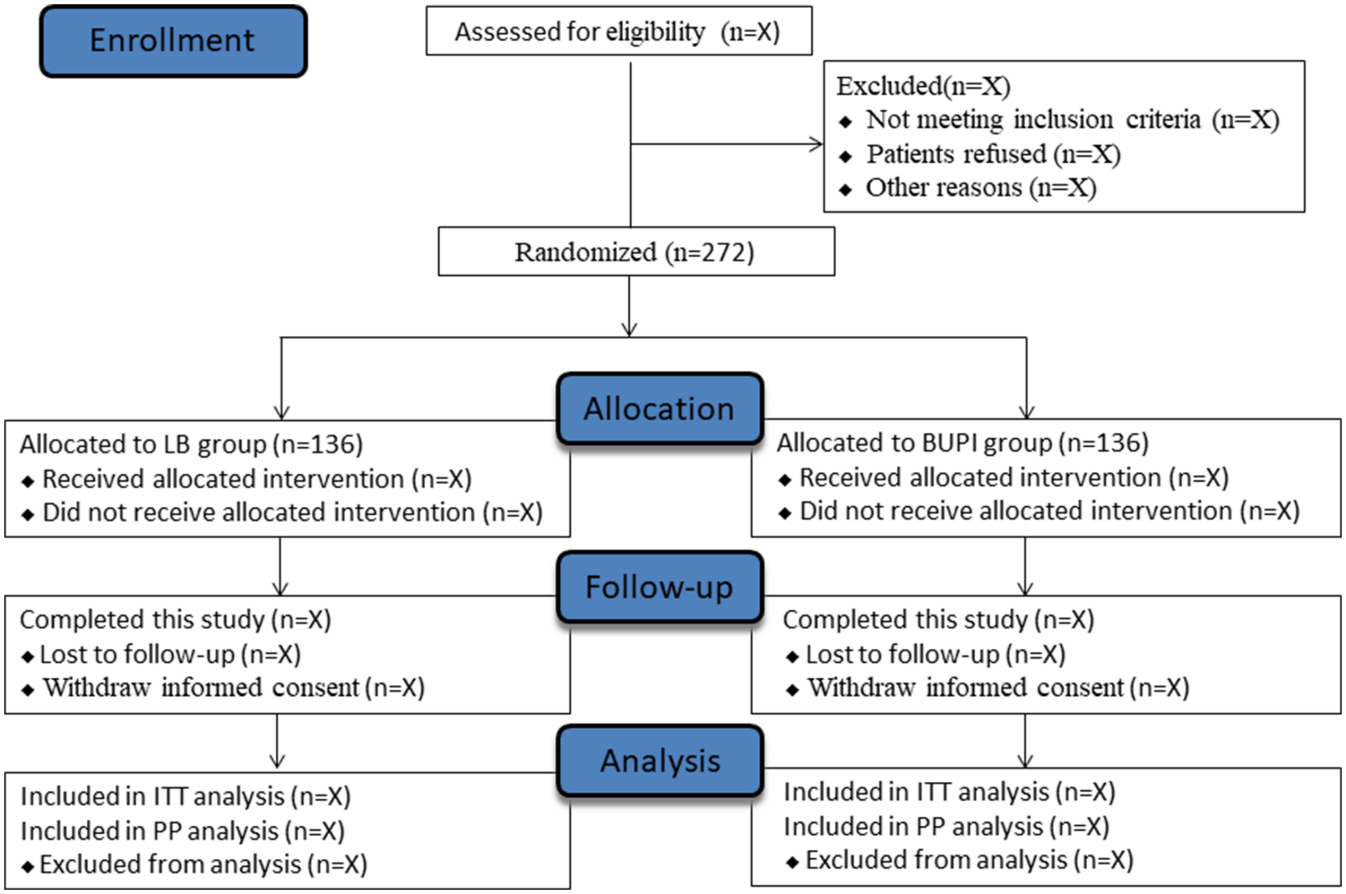
Study flow diagram.

This trial start date is 1 September 2023, and the anticipated end date is 31 March 2025. By the time of this manuscript submission, the recruitment of participants has started, but all follow-up data will be stored in the electronic data capture (EDC) system, which will be locked until the final analysis. Figure 1 shows the study flow diagram. Table 1 presents the schedule of subject enrollment, study intervention, and outcome evaluation following the SPIRIT statement.

**Table 1:** Diagrammatic representation of trial processes.

	Pre-Op (study days-6-0)	Allocation 2 h before surgery	Intra-Op (study day 1)	PACU (study day 1)	Post-Op (0–72 h) (study days 1–4)	Day of discharge	Post-Op (study days 5–8)	Post-Op (study day 15)	Post-Op 3 months
Enrollment									
Eligibility screening	╳								
Informed Consent	╳								
Demographics	╳								
Randomization		╳							
Allocation		╳							
Vital signs			╳						
Interventions									
ESPB procedure			╳						
Measurements									
Intra-operative information			╳						
NRS scoring at rest				╳	╳				
NRS scoring on activity				╳	╳			╳	╳
Opioid rescue medication					╳	╳			
Initiation of postoperative activities					╳				
QoR-15 scores					╳				
Chest tube evaluation					╳	╳	╳ (If applicable)		
Adverse events^a^			╳	╳	╳	╳	╳		
Oral analgesic medication					╳	╳	╳ (If applicable)	╳ (If applicable)	╳ (If applicable)

According to the SPIRIT statement of defining a standard protocol for clinical trials. Op, Operation; ESPB, erector spinae plane block; NRS, numeric rating scale; QoR-15 scores, Quality of Recovery 15 scores.

a including postoperative nausea and vomiting, fever, constipation, dizziness, headache, insomnia, itching, prolonged chest tube leakage, new-onset atrial fibrillation, severe ventricular arrhythmia, deep venous thrombosis, pulmonary embolism, pulmonary atelectasis, cardiac arrest, ileus, urinary retention, chylothorax, pneumothorax, and organ failure.

### Participants and enrollment

We plan to recruit eligible subjects who meet the following inclusion criteria: (1) American Society of Anesthesiologists (ASA) class I-III, (2) adult subjects who are 18–75 years of age, and (3) subjects scheduled for elective video-assisted thoracoscopic single or multi-port lung surgery under general anesthesia with bronchial intubation.

The exclusion criteria are as follows: (1) trauma and emergency subjects, (2) subjects with New York Heart Function Scale (NYHA) levels of 3–4, (3) patients with heart conduction block (sinus block or atrioventricular block), (4) patients with unstable coronary artery disease, (5) patients with gastric ulcer or gastric bleeding, (6) patients with body mass index (BMI) of <18 kg/m^2^ or > 37 kg/m^2^, (7) patients with diabetes and are being treated with insulin, (8) patients with renal dysfunction (serum creatinine values exceed normal thresholds), (9) patients with liver dysfunction (Alanine aminotransferase or Aspartate aminotransferase is more than twice the normal value), (10) subjects with coagulation dysfunction (prothrombin time or activated partial thromboplastin time is higher than the normal threshold) or patients who are taking oral anticoagulants for other medical reasons and have not stopped it before surgery, such as warfarin or new anticoagulants rivaroxaban or dabigatran, (11) subjects with alcohol abuse or heavy dependence on narcotic drugs in the last 2 months, (12) subjects with uncontrolled anxiety, schizophrenia, or other mental illness, (13) subjects who are pregnant or preparing for pregnancy, and (14) subjects with a history of allergy to local anesthetic drugs or any of the experimental medications. Written informed consent will be signed by participants before enrollment.

### Randomization and blindness

A research coordinator who would not participate in the subsequent study conducted the online randomization with a 1:1 ratio and permuted block size of 4.^1^ According to the random sequence, subjects were randomly assigned to either the liposomal bupivacaine group (LB) or the bupivacaine HCl group (BUPI). The allocation concealment was guaranteed using identical opaque sealed envelopes. An independent anesthesia nurse at each study center prepared the study medications, either liposomal bupivacaine or bupivacaine HCl, according to the random results. As liposomal bupivacaine emulsions and bupivacaine HCl have different appearances, we ensure the blinding of surgeons and operating room staff by transferring the medication to an identical opaque syringe, which is labeled with the patient’s number only. The ESPB procedures will be performed by a skilled anesthesiologist at each center who will not be involved in managing anesthesia and overseeing postoperative care. All other perioperative clinical care will be carried out following institutional standard practice. All relevant data will be recorded on the trial case report form (CRF) and entered into the EDC system within 1 week of completing the CRF forms.

### Study interventions

The drugs are prepared as follows: liposomal bupivacaine (20 mL, 266 mg) is diluted to 30 mL with 10 mL of normal saline in the LB group, while bupivacaine HCl (100 mg) is diluted to 30 mL with 10 mL of normal saline in the BUPI group. After the induction of anesthesia, an independently trained anesthesiologist performs single-injection ESPB under ultrasound guidance in the lateral position. Initially, a high-frequency linear ultrasound probe is used in a vectorial position to locate the T4 spinous process longitudinally, and then the probe is moved outward to target the T5 transverse process. Guided by planar ultrasound visualization, the nerve block operator carefully advances the needle until the top reaches the T5 transverse process bone, and then 30 mL of local anesthetic is injected slowly after the confirmation of no blood or cerebrospinal fluid with a syringe aspiration.

All subjects will receive patient-controlled intravenous analgesia (PCIA) as rescue analgesia after surgery using 100 μg of sufentanil diluted to 100 mL with normal saline (a final concentration of 1 μg/mL). The parameters for the PCIA device are as follows: no background infusion, a self-controlled bolus of 2 mL, a lockout time of 5 min, and a locking dose of 20 mL per hour. PCIA is started once the patients enter the recovery room. Patients are instructed to self-administer sufentanil using PCIA to treat pain on the numeric rating scale (NRS) score of ≥4. If the pain remains unrelieved, additional rescue medications with morphine of 2–5 mg or other opioid analgesics can be slowly injected intravenously. The PCIA is discontinued after 48 h postoperatively. Later, if subjects still experience pain (NRS scores ≥4), morphine of 5–10 mg or other opioid analgesics can be administered. Subjects in both groups receive oral celecoxib of 200 mg twice daily (at 08:00 and 16:00) as a component of the multimodal analgesic regimen from the first postsurgical day (day 2) until complete pain relief. Oral celecoxib can be used after hospital discharge if necessary.

### Perioperative management

The flow of the participants through this trial is depicted in Figure 1. Subjects do not receive sedative or analgesic medications preoperatively. In the operating room, standard monitoring includes electrocardiography, non-invasive cuff blood pressure, pulse oximetry, and temperature. The left or right radial artery is cannulated following anesthesia induction, and continuous radial artery pressure is measured. General anesthesia is induced with propofol (2–2.5 mg/kg), sufentanil (0.3–0.5 μg/kg), and cisatracurium (0.2 mg/kg) in sequence. Following tracheal intubation with a double-lumen tube, unilateral bronchial ventilation is conducted with a tidal volume of 4–6 mL/kg and a respiratory rate of 12–16 times/min, aiming to maintain the end-tidal carbon dioxide at 35–45 mmHg and pulse oxygen saturation of ≥95%. The location of the bronchial tube is then confirmed using flexible fiberoptic bronchoscopy. Anesthesia is maintained with sevoflurane inhalation targeting a bispectral index value between 40 and 60. Intraoperative analgesia is provided with sufentanil and remifentanil: sufentanil 0.1–0.2 μg/kg is given before incision at the beginning of surgery, and remifentanil (0.05–0.2 μg/kg/min) is infused continuously for intra-operative analgesia. Additional doses of sufentanil can be given intraoperatively if necessary, and sufentanil is added 0.1–0.2 μg/kg when the remifentanil is discontinued at the end of the operation. The additional doses of cisatracurium can be incremented intraoperatively as needed. Intraoperative hypotension (defined as a decrease in mean blood pressure [MBP] of >20% of baseline or MBP of <65 mmHg) would be treated with intravenous ephedrine of 6–10 mg or phenylephrine of 50–100 μg, and bradycardia (defined as heart rate [HR] of <50 beats/min) would be treated with intravenous atropine of 0.3–0.5 mg.

At the end of the surgery, all subjects received ondansetron of 8 mg as an antiemetic after the last stitches. Subjects are transferred to a post-anesthesia care unit (PACU) and kept there for at least 1 h before returning to the thoracic ward. The surgical day is regarded as the study day 1, and the day following surgical day is the study day 2.

### Postsurgical assessments

Pain intensity assessments are conducted at 0 h (~15 min after extubation), 1 h (± 10 min), 6 h (± 2 h), 12 h (± 2 h), 18 h (± 2 h), 24 h (± 2 h), 32 h (± 2 h), 40 h (± 2 h), 48 h (± 2 h), 60 h (± 2 h), and 72 h (± 2 h) postoperatively, and at patient’s request for rescue medication, where the time of the completion of surgery is considered 0 of the postoperative time. Pain intensity is assessed using an 11-point NRS (0 = no pain; 10 = worst possible pain) at rest and on activity; the latter refers to the scoring when turning over on bed if the subjects are still not out of bed for activity, or the scoring on active movement if the subjects are already out of bed. Telephone follow-up is performed if the subjects are discharged from the hospital within 72 h. During the nighttime (23:00 to 06:00, the next day), the subject is not awakened for scoring. If the subject is awake during the time window, the NRS score is assessed based on the subject’s own or companion’s recollection in the following morning; if the patient is asleep during the time window, the NRS score is standardized to a uniform value of 2. Considering the potential impact of opioid rescue medications on pain measurements, the windowed worst observation carried forward (wWOCF) method is utilized to accurately document NRS pain scores in the initial 72 h following surgery. The wWOCF approach operates in the following manner: to obtain the wWOCF for subjects who receive rescue medication, any NRS scores noted within a 2-h “window” following the administration of opioid rescue medication are replaced by the highest (“worst”) NRS scores recorded before the administration of their initial rescue medication. The time to the first rescue analgesia using PCIA was recorded, and the amount of medication used for rescue analgesia with the PCIA analgesic pump was also calculated. Quality of Recovery 15 (QoR-15) scores at 72 h after surgery are used to evaluate the subject’s recovery. NRS scores are measured once daily after 72 h postoperatively until study day 15. The number of continued days and the dosage of the oral analgesic drug celecoxib after discharge are also recorded.

### Data monitoring committee

An independent Data Monitoring Committee (DMC) comprising experienced anesthesiologists and statisticians has been established to resolve any uncertainties related to data collection and to determine whether postsurgical analgesic medications that have been administered are beyond the protocol. This DMC comprises a chair (an experienced anesthesiologist), an attending anesthesiologist, two statisticians, and an attending thoracic surgeon. When ambiguity occurs in data collection or in adherence to protocol medications, these issues are discussed at the DMC to reach a final conclusion.

### Trial outcome definitions

#### Primary outcome

The primary outcome for the trial is the area under the curve of pain scores at rest for 0–72 h (AUC NRS-R_0-72_) postoperatively.

#### Secondary outcomes

The secondary outcomes include (1) the total amount of opioid rescue analgesics through 0–72 h postoperatively, (2) the time to first press on the PCIA device as rescue analgesia, (3) the area under the curve of pain scores on activity for 0–72 h (AUC NRS-A_0-72_) postoperatively, (4) NRS scores at rest and on the activity at different time points during the 0–72 h postoperative period, (5) Quality of Recovery 15 (QoR-15) scores at 72 h after surgery, (6) NRS scores on activity on postsurgical day 14 (study day 15), which will be followed up via telephone, with a permitted time window of 14 ± 3 days, and (7) NRS scores on the activity at postsurgical 3 months will be followed up via telephone, with a permitted time window of 3 months ±7 days. Opioids are first converted to intravenous morphine equivalents for each subject, and then the total amount will be naturally logarithmically converted before analysis. The time to first opioid rescue analgesia using PCIA was measured in hours, i.e., the date and time of the first opioid rescue minus the date and time of the end of surgery.

#### Tertiary outcomes

Tertiary outcomes include (1) the time to initiation of postoperative activities around the bed, (2) the total volume of chest tube drainage, (3) the time to chest tube removal, (4) the duration of oral analgesic medications taken by the subject postoperatively in the telephone follow-up, and (5) the total amount of oral analgesic medications taken postoperatively. The total volume of chest tube drainage is referred to as the total amount of chest tube drainage until the chest tube removal. The time to initiate postoperative activities around the bed and time to chest tube removal are measured in hours, i.e., the time of the event minus the time of the end of surgery.

#### Safety outcomes

Safety outcomes are assessed separately and then combined for all subjects, consisting of adverse events during and after surgery. The incidence of adverse events after the surgery is tracked up to the 7th postsurgical day (study day 8). Adverse events include postoperative nausea and vomiting (PONV), fever, constipation, dizziness, headache, insomnia, itching, prolonged chest tube leakage, new-onset atrial fibrillation, severe ventricular arrhythmia, deep venous thrombosis, pulmonary embolism, pulmonary atelectasis, cardiac arrest, ileus, urinary retention, chylothorax, pneumothorax, and organ failure. The definitions of adverse events are displayed in the Supplementary Table S1. Any adverse event in relation to the study interventions must be reported to DMC in the form of an “Adverse Event Form” within 24 h. In the event of a serious adverse event, such as an unexpected deterioration in the patient’s clinical condition during the perioperative period, the attending anesthesiologist could require unblinding and adjustment or discontinuation of administration.

### Sample size calculation

Between January 2023 and March 2023, we conducted a prospective study on two groups of 10 patients each. All participants underwent video-assisted thoracoscopic lung surgery and were administered a preoperative ultrasound-guided ESPB, with one group receiving liposome bupivacaine and the other bupivacaine HCl. The results showed that the postoperative AUC NRS-R0-72 was 247 ± 35 in the LB group versus 262 ± 43 in the BUPI group. Based on the preliminary study, the sample size was determined to ensure the ability to detect a mean difference of 15 in the primary efficacy outcome, and we suggest that this difference is clinically relevant. This calculation is performed with a two-group *t*-test, a standard deviation of 38.7, an 80% power, and a 0.05 level of significance. Considering a 20% dropout rate, this study will enroll a total of 272 patients, with 136 in each group. The sample size is calculated using the SAS software (version 9.4, SAS Institute Inc).

### Statistical analysis

Baseline characteristics will be tabulated using applicable summary statistics. Outcome data will be analyzed according to the intention to treat (ITT) principle and secondarily with the per-protocol (PP) analysis.

The ITT population will comprise all randomized patients who receive ultrasound-guided ESPB procedures and complete the surgery with the primary outcome measurement available. These subjects will be analyzed according to the groups to which they are randomized, regardless of whether the surgical procedure is converted to an open thoracotomy or any other surgical procedure implemented within 3 months postoperatively. Subjects who are given non-protocol pain medication postoperatively will also be included in the ITT analysis. Subjects whose consent is withdrawn will have their data retained until the time of withdrawal. The PP population will comprise those patients who complete the study based on the original protocol. Subjects with modifications of the original surgical approach to open thoracotomy or who undergo any other surgical procedures within 3 months after surgery are specifically excluded from this analysis. Data from subjects whose consent is withdrawn will be used until the time of withdrawal of consent.

Demographic information and baseline characteristics will be described using descriptive statistics only, with no between-group comparisons. Continuous data will be displayed as means and standard deviations or medians and interquartile ranges, depending on data distribution. Categorical data will be summarized as counts and percentages. Between-group differences of continuous data will be analyzed using the independent t-test or Mann–Whitney rank sum test as appropriate, while categorical data will be analyzed using the Chi-squared test or Fisher’s exact test as appropriate. To analyze the treatment effect, odds ratios are calculated for binary data and mean differences for continuous data, each together with their 95% confidence intervals. For the primary outcomes, the between-group difference of AUC NRS-R_0-72_ is regarded as significant at the two-side *p*-value of 0.05. For the secondary and tertiary outcomes, multiple comparisons are adjusted for the significance level of probability values by computing the false discovery rate using the Benjamini-Hochberg method. To further understand the cumulative effect of study interventions on postsurgical pain, *post-hoc* analysis is conducted, including AUC NRS-R_0-24_, AUC NRS-R_24-48_, AUC NRS-R_48-72_, AUC NRS-A_0-24_, AUC NRS-A_24-48_, and AUC NRS-A_48-72_, the cumulative amount of opioid rescue analgesics during 0–24, 24–48, and 48––72 h, and the proportion of subjects who are pain-free (NRS-R score of 0 or 1) at 1, 6, 24, 48, and 72 h. To address the missing NRS pain intensity scores during the postoperative 0–72 h, interpolation is performed using one of the following methods: (1) by last observation carried forward (LOCF) imputation method if missing data are located after the last non-missing score, (2) by linear interpolation if more than one missing data occur and are located between two non-missing scores (25). All data will be analyzed using the SAS software (version 9.4, SAS Institute Inc) by an independent statistician.

## Discussion

This multicenter, randomized, double-blind, parallel-controlled trial will include 272 subjects who will undergo elective video-assisted thoracoscopic lung surgery. We will determine the effect of LB versus bupivacaine HCl for preoperative ultrasound-guided ESPB on AUC NRS-R_0-72_, the amount of opioid rescue analgesics after surgery, time to the first rescue analgesia, AUC NRS-A_0-72_, NRS scores at rest and on activity at different time points during the 0–72 h postoperative period, and NRS scores on activity on postsurgical day 14 and postsurgical 3 months. Our primary hypothesis is that the use of LB for ultrasound-guided ESPB preemptive analgesia provides a lower area under the curve of pain scores at rest 0–72 h postoperatively compared to bupivacaine HCl. Moreover, we will explore the adverse events associated with the application of LB for ultrasound-guided ESPB. The administration of this trial and the presentation of results will follow the Consolidated Standards of Reporting Trials guidelines.

Regional nerve block maneuvers before skin incisions are considered an optimal method of preemptive analgesia, which facilitates the reduction of postsurgical pain. ESPB is a novel interfascial regional anesthesia technique first introduced by Forero et al. in 2016 for thoracic neuropathic pain treatment and has rapidly gained prevalence in a diverse range of surgical procedures (7, 26). In particular, it is easy to implement owing to the simple identification of anatomic landmarks on ultrasound and the fact that no vital organs are nearby (27). Anatomical and radiological investigations in fresh cadavers have suggested that the clinical effects of ESPB are likely derived from the theoretical anterior distribution of the local anesthetics through the intertransverse connective tissue or the costotransverse foramen to infiltrate the ventral crus of the spinal nerves, the dorsal root ganglion, and the sympathetic chain, thereby resulting in an effect similar to epidural analgesia (7, 28).

Recent studies have demonstrated that ESPB is a simple, safe, wide-ranging, and efficacious alternative analgesic technique for postsurgical pain (7–9, 29). A recent meta-analysis including 14 studies indicated that ESPB significantly lowered pain scores at rest or on movement, decreased 24 h opioid consumption, and reduced the incidence of postoperative nausea and vomiting compared with the non-block care in breast and thoracic surgery (30). Another meta-analysis also suggested that ultrasound-guided ESPB could provide an opioid-sparing effect in subjects undergoing surgeries with general anesthesia, thereby reducing the adverse events related to opioid administration, such as nausea, vomiting, and delayed peristalsis (29, 31, 32). The currently prevalent regional nerve block maneuvers in thoracoscopic surgery involve paravertebral and intercostal nerve blocks. Each of these procedures has its unique strengths and inherent disadvantages. For instance, paravertebral block is performed close to the spinal canal and vascular plexus, with technical complexity and potential risk of serious complications, while the effectiveness of intercostal nerve block may be limited to a dermatomal extent (33). Given the combination of its efficacy and lower associated risks, ESPB has been preferred in our study.

Liposomal bupivacaine (Bupivacaine Liposome Injection; Jiangsu Hengrui Pharmaceuticals Co., Ltd.; China) is a novel liposome-encapsulated local anesthetic used for surgical site administration to yield postsurgical analgesia. The delivery of local anesthetics using encapsulating agents is a desirable alternative, as it provides a system for sustained release and subsequently enhances analgesia (34). Liposomal bupivacaine has been shown to provide postsurgical analgesia with a similar safety profile to bupivacaine HCl in a variety of surgical scenarios, such as hemorrhoidectomy, total knee arthroplasty, mammoplasty, and thoracic surgery (18, 35–37). Liposome bupivacaine appears safe when used in fascial plane blocks and peripheral nerve blocks, such as ESPB, paravertebral block, and intercostal nerve block (16–20, 23).

However, evidence of the clinical effectiveness of interfascially or perineurally applied liposomal bupivacaine in extending the duration of postoperative analgesia of nerve blocks is insufficient. Several retrospective studies have demonstrated the superiority of LB over bupivacaine HCl in controlling postsurgical pain when the paravertebral blocks or intercostal nerve blocks are applied in thoracic surgery (21–23). Another retrospective study included 387 patients receiving either liposomal bupivacaine for an intrathoracic intercostal nerve block or thoracic epidural bupivacaine HCl in video-assisted thoracoscopic surgery (38). Liposomal bupivacaine used for the regional block was comparable with bupivacaine HCl for epidural analgesia, taking into account healthcare costs and analgesia efficacy. A randomized controlled trial compared the analgesic efficiency and safety of single-dose transversus abdominis plane (TAP) blocks with liposomal bupivacaine and sustained epidural analgesia, with bupivacaine HCl in 498 subjects undergoing major abdominal surgery (39). The results showed that pain scores at rest during the initial postsurgical days were similar between groups. Compared to subjects receiving epidural analgesia with bupivacaine HCl, those who received TAP blocks with liposomal bupivacaine required more opioid medication but had fewer complications, such as hypotension. Another randomized controlled study with a non-inferiority design that enrolled 112 patients undergoing ambulatory shoulder surgery demonstrated that interscalene nerve blocks with perineural LB offered similarly effective analgesia as the perineural standard bupivacaine with dexamethasone (40). A recent meta-analysis also suggested that perineural liposomal bupivacaine provided a statistically significant but clinically unconsidered improvement in the area under the curve of postoperative pain scores compared to plain local anesthetic (41, 42).

The variations in study protocols and injection site locations could influence study results. The pharmacokinetics profile of liposomal bupivacaine is apparently related to the duration of regional blocks (43). Liposomal bupivacaine exhibits a biphasic model, dose-related release profile with an initial peak release within 1 h of execution related to extra liposomal bupivacaine included in every ampule, followed by a further peak 12 to 48 h later, associated with the release from the liposomes (13, 44). Hence, preemptive analgesia with liposomal bupivacaine has the potential to reduce postoperative pain intensity. Furthermore, the rate of bupivacaine release from liposomes is speculated to be related to the vascularity of the surrounding tissue at the injection site. For example, approximately 30% of bupivacaine is released during the first 24 h when it infiltrates directly into tissues in knee replacement, and approximately 90% in more vascularly distributed hemorrhoidectomy (45). The erector spinae plane does not have a rich vascular plexus, and the rate of bupivacaine release from liposomes may be similar to other fascial plane blocks, allowing for prolonged anesthetic duration. More studies are warranted to determine the exact features and effectiveness of this block in various surgical procedures. To date, this is the first randomized controlled study to determine the efficiency of liposomal bupivacaine for ESPB on postoperative pain in thoracic surgery.

In this study, we formulate certain rules for NRS scoring to achieve multicenter consistency and eliminate the influence of external factors on pain outcomes. For instance, we have implemented the wWOCF protocol for recording NRS pain intensity scores in the first 72 h after surgery. This method is designed to reduce the influence of opioid rescue medications on the NRS evaluations. Specifically, if patients receive rescue medication within a 2 h “window” before an assessment, we will replace the score for that time point with the highest (“worst”) NRS score recorded before the administration of the initial rescue medication. In addition, we employ a nocturnal recall score to minimize the disruption to the patient’s nighttime rest. We do not take bedside face-to-face scoring at night (23:00 to 06:00, the following day). If the subject is awake within the time window, the subject or companion recollects the pain state the following morning, and if the subject is asleep during the time window, the NRS score is assigned a uniform value of 2.

Our study has several limitations. First, partial subjects were discharged within 72 h after surgery, and NRS scores for these subjects could only be followed up by telephone. Second, adverse events are only tracked up to the 7th postsurgical day without further follow-up. Finally, varying durations of oral analgesic medications or non-protocol analgesics may interfere with the NRS scoring for pain.

In conclusion, this randomized clinical trial was designed to determine the effect of liposomal bupivacaine versus bupivacaine HCl for preoperative ultrasound-guided ESPB on postoperative pain in video-assisted thoracoscopic lung surgery. We expected that liposomal bupivacaine could be safely employed in ESPB, and liposomal bupivacaine for preoperative ultrasound-guided ESPB would be more effective than bupivacaine HCl in reducing the area under the curve of pain scores at rest from 0 to 72 h postoperatively.

## Ethics statement

The studies involving humans were approved by the Medical Ethics Committee of the First Affiliated Hospital of Soochow University. The studies were conducted in accordance with the local legislation and institutional requirements. Written informed consent for participation in this study was provided by the participants' legal guardians/next of kin. Written informed consent was obtained from the individual(s) for the publication of any potentially identifiable images or data included in this article.

## Author contributions

DL: Data curation, Project administration, Writing – original draft, Writing – review & editing. KP: Conceptualization, Investigation, Methodology, Supervision, Writing – review & editing. YZ: Data curation, Formal analysis, Methodology, Project administration, Writing – review & editing. HuL: Conceptualization, Data curation, Investigation, Software, Writing – review & editing. ZX: Conceptualization, Project administration, Writing – review & editing. JG: Conceptualization, Project administration, Writing – review & editing. FW: Conceptualization, Project administration, Writing – review & editing. CC: Conceptualization, Project administration, Writing – review & editing. XLv: Conceptualization, Project administration, Writing – review & editing. JT: Conceptualization, Project administration, Writing – review & editing. XLi: Conceptualization, Project administration, Writing – review & editing. XQ: Conceptualization, Project administration, Writing – review & editing. XW: Conceptualization, Project administration, Writing – review & editing. YW: Conceptualization, Project administration, Writing – review & editing. SO: Conceptualization, Project administration, Writing – review & editing. HoL: Supervision, Validation, Writing – review & editing. XS: Conceptualization, Project administration, Writing – original draft, Writing – review & editing. FJ: Conceptualization, Funding acquisition, Resources, Supervision, Writing – review & editing.

## Footnotes

^1^ http://random.91trial.com
