# Supplementary material for: Effect of liposomal bupivacaine for preoperative erector spinae plane block on postoperative pain following video-assisted thoracoscopic lung surgery: a protocol for a multicenter, randomized, double-blind, clinical trial
Source: Front Med (Lausanne). 2024 Apr 12;11:1359878. doi: 10.3389/fmed.2024.1359878 (PMC11045961; doi:10.3389/fmed.2024.1359878)
Supplement: Supplementary file 1 [file Table_1.docx]

**Table S1. Definitions of adverse events within 7 days after surgery according to the ACS NSQIP**

| **Event** | **Definition** |
| --- | --- |
| PONV | Occurring nausea or vomiting within 24 hours after surgery. |
| Fever | An ear temperature higher than 38 ℃ |
| Constipation | Difficulty with defecation and complaints of hard stools or straining. We also describe constipation as fewer than 3 defecations per week |
| Dizziness | A common functional cerebral disorder. Feeling dizzy within 7 days after surgery. |
| Headache | Feeling intermittent or persistent headache within 7 days after surgery. |
| Insomnia | Difficulty with sleep. Insomnia will be used as a disorder with the following diagnostic criteria: (1) difficulty falling asleep, staying asleep or nonrestorative sleep; (2) this difficulty is present despite adequate opportunity and circumstance to sleep; (3) this impairment in sleep is associated with daytime impairment or distress; and (4) this sleep difficulty occurs at least 3 times per week and has been a problem for at least 1 month. |
| Itching | Itching sensation at the site of drug injection or all over the body |
| Prolonged chest tube leakage | Persistent chest tube leak for more than 5 days. |
| New-onset atrial fibrillation | Indicate whether the patient had a new onset of atrial fibrillation (AF) requiring treatment. Does not include recurrence of previously documented AF which had been present preoperatively. |
| Severe ventricular arrhythmia | Life-threatening ventricular arrhythmias, including ventricular tachycardia or ventricular fibrillation. |
| Deep venous thrombosis | A new blood clot or thrombus within the venous system. |
| Pulmonary embolism | Lodging of a blood clot in the pulmonary artery with subsequent obstruction of blood supply to the lung parenchyma. The identification of a new blood clot in a pulmonary artery causing obstruction (complete or partial) of the blood supply to the lungs. A pulmonary embolism must be noted within 30 days after the principal operative procedure and the following criteria, A and B below: A. New diagnosis of a new blood clot in a pulmonary artery and B. The patient has a V-Q scan interpreted as high probability of pulmonary embolism or a positive CT exam, TEE, pulmonary arteriogram, CT angiogram, or any other definitive imaging modality (including direct pathology examination such as autopsy. |
| Pulmonary atelectasis | Decrease in the volume or air content of one or more lung segments or lobes. Pulmonary atelectasis in our study is defined as chest CT diagnosis indicating atelectasis. |
| Cardiac arrest | The absence of cardiac rhythm or presence of chaotic cardiac rhythm, intraoperatively or within 30 days following surgery, which results in a cardiac arrest requiring the initiation of CPR, which includes chest compressions. Patients are included who are in a pulseless VT or VF in which defibrillation is performed and PEA arrests requiring chest compressions. Patients with AICD that fire but the patient has no loss of consciousness are excluded. |
| Ileus | Obstruction of passage of intestinal contents from any cause. Diagnosis can be based on the four major symptoms of abdominal pain, vomiting, abdominal distension, and cessation of defecation. Visible bowel pattern or peristalsis in the abdomen waves and hyperactive bowel sound are also typical clinical symptom. |
| Urinary retention | Acute or chronic inability to voluntarily pass an adequate amount of urine. Acute urinary retention is a urologic emergency characterized by the sudden inability to urinate combined with suprapubic pain, bloating, urgency, distress, or, occasionally, mild incontinence. Chronic urinary retention refers to postvoid residual volume greater than 300 ml measured on two separate occasions and persisting for at least six months. |
| Chylothorax | Various reasons result in the leakage of lymphatic chylous fluid flowing back through the thoracic duct and its accumulation in the pleural cavity. Chylothorax is associated with thoracic duct injury or occlusion. |
| Pneumothorax | The accumulation of air in the pleural space. Pneumothorax is divided into two main categories: traumatic and spontaneous. Traumatic pneumothorax may be due to blunt or penetrating trauma or may be iatrogenic from medical procedures associated with visceral pleural injury. Spontaneous pneumothorax has been divided into primary (ie, in the absence of underlying lung disease) and secondary (ie, associated with underlying lung disease) etiologies. Pneumothorax is diagnosed by chest CT or X-ray. |
| Organ failure | Some pathological factors result in serious dysfunction or decompensation of one or more organs, which is manifested as a certain clinical syndrome. |

ACS NSQIP, the National Surgical Quality Improvement Program from the American College of Surgeons; PONV, postoperative nausea and vomiting; AF, atrial fibrillation; CT, computerized tomography; TEE, trans-esophageal echocardiography; VT, ventricular tachycardia; VF, ventricular fibrillation; CPR, cardiopulmonary resuscitation; PEA, pulseless electrical activity; AICD, automated implantable cardioverter defibrillator.
